# *N*-(2,4-Difluoro­phen­yl)-5-methyl-1,2-oxazole-4-carboxamide hemihydrate

**DOI:** 10.1107/S1600536812029431

**Published:** 2012-07-04

**Authors:** Jian-Guang Yu, Hai-Xi Zhu, Jiang-Kai Qiu, De-Cai Wang, Hong Xu

**Affiliations:** a State Key Laboratory of Materials-Oriented Chemical Engineering, School of Pharmaceutical Sciences, Nanjing University of Technology, Xinmofan Road No. 5 Nanjing, Nanjing 210009, People’s Republic of China; b State Key Laboratory of Materials-Oriented Chemical Engineering, College of Food Science and Light Industry, Nanjing University of Technology, Xinmofan Road No. 5 Nanjing, Nanjing 210009, People’s Republic of China

## Abstract

In the title compound, C_11_H_8_F_2_N_2_O_2_·0.5H_2_O, the dihedral angle between the benzene and isoxazole rings is 8.08 (3)°. In the crystal, the components are linked by O—H⋯N and N—H⋯O hydrogen bonds, in which the water mol­ecule acts as both a donor and an acceptor, into a tape with an *R*_4_^4^(16) graph-set motif along the *a* axis. The water mol­ecule is located on a twofold rotation axis. The methyl H atoms were treated as disordered groups over two sites with a refined site-occupancy ratio of 0.48 (6):0.52 (6).

## Related literature

For applications of leflunomide [systematic name: 5-methyl-*N*-[4-(trifluoro­meth­yl) phen­yl]-isoxazole-4-carboxamide] in the treatment of rheumatoid arthritis, see: Shaw *et al.* (2011 ▶); Schattenkirchner (2000 ▶); For leflunomide analogs, see: Huang *et al.* (2003 ▶). For graph-set motifs, see: Bernstein, *et al.* (1995 ▶).
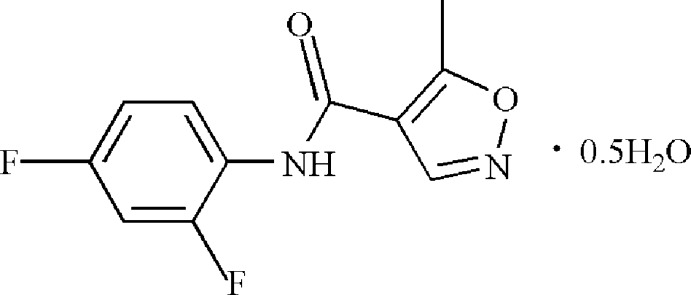


## Experimental

### 

#### Crystal data

C_11_H_8_F_2_N_2_O_2_·0.5H_2_O
*M* *_r_* = 247.20
Monoclinic, 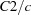

*a* = 15.182 (3) Å
*b* = 13.803 (3) Å
*c* = 12.159 (2) Å
β = 120.06 (3)°
*V* = 2205.3 (8) Å^3^
*Z* = 8
Mo *K*α radiation
μ = 0.13 mm^−1^
*T* = 293 K
0.30 × 0.20 × 0.10 mm

#### Data collection

Enraf–Nonius CAD-4 diffractometer
Absorption correction: multi-scan (North *et al.*, 1968 ▶) *T*_min_ = 0.962, *T*_max_ = 0.987
2076 measured reflections
1997 independent reflections
1486 reflections with *I* > 2σ(*I*)
*R*_int_ = 0.050
3 standard reflections every 200 reflections intensity decay: 1%

#### Refinement

*R*[*F* ^2^ > 2σ(*F* ^2^)] = 0.050
*wR*(*F* ^2^) = 0.130
*S* = 1.04
1997 reflections
168 parameters
H atoms treated by a mixture of independent and constrained refinement
Δρ_max_ = 0.28 e Å^−3^
Δρ_min_ = −0.18 e Å^−3^

### 

Data collection: *CAD-4 EXPRESS* (Enraf–Nonius, 1994 ▶); cell refinement: *CAD-4 EXPRESS*; data reduction: *XCAD4* (Harms & Wocadlo, 1995 ▶); program(s) used to solve structure: *SHELXS97* (Sheldrick, 2008 ▶); program(s) used to refine structure: *SHELXL97* (Sheldrick, 2008 ▶); molecular graphics: *SHELXTL* (Sheldrick, 2008 ▶); software used to prepare material for publication: *SHELXTL*.

## Supplementary Material

Crystal structure: contains datablock(s) I, global. DOI: 10.1107/S1600536812029431/nk2167sup1.cif

Structure factors: contains datablock(s) I. DOI: 10.1107/S1600536812029431/nk2167Isup2.hkl

Supplementary material file. DOI: 10.1107/S1600536812029431/nk2167Isup3.cml

Additional supplementary materials:  crystallographic information; 3D view; checkCIF report

## Figures and Tables

**Table 1:** Hydrogen-bond geometry (Å, °)

*D*—H⋯*A*	*D*—H	H⋯*A*	*D*⋯*A*	*D*—H⋯*A*
N1—H1*A*⋯O1*W*	0.88 (3)	2.26 (3)	3.052 (3)	150 (2)
O1*W*—H1*W*⋯N2^i^	0.92 (3)	2.03 (3)	2.934 (2)	167 (3)

Symmetry code: (i) 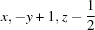.

**Fig. 1.**
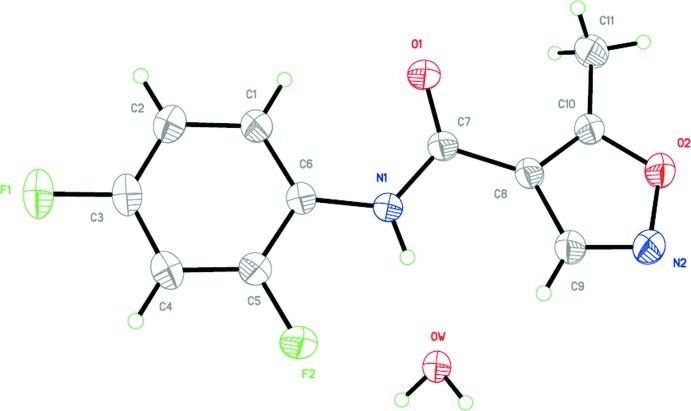
The structure of the title compound, showing the atomic numbering scheme. Non-H atoms are shown with 30% probability displacement ellipsoids.

## supplementary crystallographic information

### Comment

Leflunomide is one of the most effective isoxazole-containing disease-modifying drugs for treating rheumatoid arthritis (Shaw, *et al.*, 2011; Schattenkirchner, 2000). Many leflunomide analogs have been synthesized and exhibit potent immunomodulating effect (Huang, *et al.*, 2003). The title compound, N-(2,4-difluorophenyl)-5-methylisoxazole-4-carboxamide monohydrate, was synthesized as a novel and potent immunomodulating leflunomide analog. We report herein its crystal structure.

As illustrated in Fig. 1, the molecular structure of the title compound is not planar and consists of one N-(2,4-difluorophenyl)-5-methylisoxazole-4-carboxamide molecule and one solvate water molecule. The C1-C6 benzene and the C8-C10/N2/O2 isoxazole ring is almost coplannar with each other with the dihedral angle of 8.08 (3) °. The central nitrogen atom (N1) and carbon atom (C7) are nearly coplanar with the benzene ring and the isoxazole rings[N1-C6-C5-C4 torsion angles = -178.8 (2) ° and C7-C8-C10-O2 torsion angles = 177.4 (2) °], respectively. The length of the C9=N2 double bond is 1.296 (3) Å, slightly longer than standard 1.28 Å value of a C=N double bond. The crystal structure is stabilized by O—H···N and N—H···O hydrogen bonds among the solvate water molecules, amide group and isoxazole nitrogen atoms (Table 1). It is noted that the connections of water molecules and N-(2,4-difluorophenyl)-5-methylisoxazole-4-carboxamide molecules generate a tape with R^4^_4_(16) pattern based on graph set analysis nomenclature (Bernstein, *et al.*, 1995).

### Experimental

A solution of 0.005 mole of 5-methylisoxazole-4-carboxylic acid chloride (0.73 g) in 2 ml of acetonitrile was added drop-wise, while stirring, to 0.01 mole of 2,4-difluoroaniline (1.29 g),dissolved in 15 ml of acetonitrile at room temperature. After stirring for 20 minutes, the precipitated 2,4-difluoroaniline hydrochloride was filtered off and washed with 10 ml portions of acetonitrile, and the combined filtrates were concentrated under reduced pressure. 8.7g (73.10% yield) of white crystalline N-(2,4-difluorophenyl)-5-methylisoxazole-4-carboxamide were thus obtained. Crystals of the title compound suitable for X-ray diffraction were obtained by slow evaporation of a toluene solution.

### Refinement

H atoms of the water molecule were located in a difference Fourier map and refined as riding with O—H = 0.85 Å, with *U*_iso_(H) = 1.5 *U*_eq_. Carbon and nitrogen bound H atoms were placed at calculated positions and were treated as riding on the parent C or N atoms with C—H = 0.96 (methyl), 0.93 (methylene) and N—H = 0.88 Å, *U*_iso_(H) = 1.2 or 1.5 *U*_eq_(C, N). The methyl H atoms are treated as disordered groups over two sites with a refined site-occupancy ratio of 0.48: 0.52 (6). The hydrogen atoms involving the hydrogen bonding interaction perform not well when freely refined and it can not provide a reasonable basis for discussion of hydrogen bonding interaction

### Crystal data

**Table:**

C_11_H_8_F_2_N_2_O_2_·0.5H_2_O	*F*(000) = 1016
*M**_r_* = 247.20	*D*_x_ = 1.489 Mg m^−^^3^
Monoclinic, *C*2/*c*	Mo *K*α radiation, λ = 0.71073 Å
Hall symbol: -C 2yc	Cell parameters from 25 reflections
*a* = 15.182 (3) Å	θ = 9–13°
*b* = 13.803 (3) Å	µ = 0.13 mm^−^^1^
*c* = 12.159 (2) Å	*T* = 293 K
β = 120.06 (3)°	Block, white
*V* = 2205.3 (8) Å^3^	0.30 × 0.20 × 0.10 mm
*Z* = 8	

### Data collection

**Table:**

Enraf–Nonius CAD-4 diffractometer	1486 reflections with *I* > 2σ(*I*)
Radiation source: fine-focus sealed tube	*R*_int_ = 0.050
Graphite monochromator	θ_max_ = 25.2°, θ_min_ = 2.1°
ω/2θ scans	*h* = 0→18
Absorption correction: multi-scan (North *et al.*, 1968)	*k* = 0→16
*T*_min_ = 0.962, *T*_max_ = 0.987	*l* = −14→12
2076 measured reflections	3 standard reflections every 200 reflections
1997 independent reflections	intensity decay: 1%

### Refinement

**Table:**

Refinement on *F*^2^	Secondary atom site location: difference Fourier map
Least-squares matrix: full	Hydrogen site location: inferred from neighbouring sites
*R*[*F*^2^ > 2σ(*F*^2^)] = 0.050	H atoms treated by a mixture of independent and constrained refinement
*wR*(*F*^2^) = 0.130	*w* = 1/[σ^2^(*F*_o_^2^) + (0.060*P*)^2^ + 1.6*P*] where *P* = (*F*_o_^2^ + 2*F*_c_^2^)/3
*S* = 1.04	(Δ/σ)_max_ < 0.001
1997 reflections	Δρ_max_ = 0.28 e Å^−^^3^
168 parameters	Δρ_min_ = −0.18 e Å^−^^3^
0 restraints	Extinction correction: *SHELXL97* (Sheldrick, 2008), Fc^*^=kFc[1+0.001xFc^2^λ^3^/sin(2θ)]^-1/4^
Primary atom site location: structure-invariant direct methods	Extinction coefficient: 0.0247 (19)

### Special details

**Table:**

Geometry. All esds (except the esd in the dihedral angle between two l.s. planes) are estimated using the full covariance matrix. The cell esds are taken into account individually in the estimation of esds in distances, angles and torsion angles; correlations between esds in cell parameters are only used when they are defined by crystal symmetry. An approximate (isotropic) treatment of cell esds is used for estimating esds involving l.s. planes.
Refinement. Refinement of F^2^ against ALL reflections. The weighted R-factor wR and goodness of fit S are based on F^2^, conventional R-factors R are based on F, with F set to zero for negative F^2^. The threshold expression of F^2^ > 2sigma(F^2^) is used only for calculating R-factors(gt) etc. and is not relevant to the choice of reflections for refinement. R-factors based on F^2^ are statistically about twice as large as those based on F, and R- factors based on ALL data will be even larger.

### Fractional atomic coordinates and isotropic or equivalent isotropic displacement parameters (Å^2^)

**Table:**

	*x*	*y*	*z*	*U*_iso_*/*U*_eq_	Occ. (<1)
O1W	0.5000	0.52321 (17)	0.2500	0.0620 (7)	
H1W	0.505 (2)	0.564 (2)	0.193 (3)	0.093*	
N1	0.65863 (15)	0.36286 (13)	0.32726 (18)	0.0484 (5)	
H1A	0.6314 (18)	0.4180 (19)	0.332 (2)	0.058*	
O1	0.66094 (13)	0.20060 (11)	0.36060 (16)	0.0611 (5)	
F1	0.88573 (17)	0.40738 (15)	0.0910 (2)	0.1141 (8)	
C1	0.75004 (19)	0.28877 (17)	0.2259 (2)	0.0584 (6)	
H1B	0.7336	0.2263	0.2381	0.070*	
F2	0.71615 (12)	0.53616 (10)	0.28913 (16)	0.0739 (5)	
O2	0.49066 (13)	0.27282 (12)	0.53567 (16)	0.0615 (5)	
N2	0.51344 (18)	0.37236 (15)	0.5487 (2)	0.0658 (6)	
C2	0.8072 (2)	0.3024 (2)	0.1673 (3)	0.0720 (8)	
H2B	0.8293	0.2495	0.1403	0.086*	
C3	0.8304 (2)	0.3937 (2)	0.1496 (3)	0.0720 (8)	
C4	0.80009 (19)	0.47406 (19)	0.1878 (2)	0.0649 (7)	
H4A	0.8162	0.5362	0.1742	0.078*	
C5	0.74512 (18)	0.45851 (16)	0.2468 (2)	0.0530 (6)	
C6	0.71701 (16)	0.36750 (15)	0.2668 (2)	0.0461 (5)	
C7	0.63608 (16)	0.28191 (15)	0.37245 (19)	0.0454 (5)	
C8	0.57905 (16)	0.30012 (15)	0.43950 (19)	0.0450 (5)	
C9	0.56469 (19)	0.38588 (17)	0.4907 (2)	0.0556 (6)	
H9A	0.5893	0.4460	0.4838	0.067*	
C10	0.53140 (17)	0.23129 (16)	0.4714 (2)	0.0492 (6)	
C11	0.5167 (2)	0.12579 (18)	0.4509 (3)	0.0674 (7)	
H11A	0.4739	0.1028	0.4827	0.101*	0.48 (6)
H11B	0.5815	0.0938	0.4950	0.101*	0.48 (6)
H11C	0.4850	0.1121	0.3617	0.101*	0.48 (6)
H11D	0.5423	0.1049	0.3970	0.101*	0.52 (6)
H11E	0.4454	0.1109	0.4112	0.101*	0.52 (6)
H11F	0.5526	0.0929	0.5311	0.101*	0.52 (6)

### Atomic displacement parameters (Å^2^)

**Table:**

	*U*^11^	*U*^22^	*U*^33^	*U*^12^	*U*^13^	*U*^23^
O1W	0.0925 (18)	0.0490 (13)	0.0742 (16)	0.000	0.0640 (15)	0.000
N1	0.0634 (12)	0.0366 (9)	0.0596 (11)	0.0022 (8)	0.0415 (10)	0.0026 (8)
O1	0.0854 (12)	0.0377 (9)	0.0823 (12)	0.0037 (8)	0.0584 (10)	0.0019 (8)
F1	0.1517 (18)	0.1052 (14)	0.1635 (19)	0.0058 (12)	0.1374 (17)	0.0148 (13)
C1	0.0760 (16)	0.0463 (12)	0.0695 (15)	−0.0001 (11)	0.0489 (14)	0.0005 (11)
F2	0.0977 (11)	0.0427 (8)	0.1108 (12)	−0.0031 (7)	0.0743 (10)	−0.0029 (7)
O2	0.0787 (11)	0.0558 (10)	0.0700 (11)	−0.0022 (8)	0.0522 (9)	0.0004 (8)
N2	0.0953 (16)	0.0509 (12)	0.0720 (14)	−0.0040 (11)	0.0574 (14)	−0.0064 (10)
C2	0.092 (2)	0.0656 (17)	0.0854 (18)	0.0076 (14)	0.0646 (17)	0.0003 (14)
C3	0.0829 (19)	0.0760 (18)	0.0865 (19)	−0.0005 (15)	0.0644 (17)	0.0076 (15)
C4	0.0736 (17)	0.0581 (15)	0.0808 (18)	−0.0062 (13)	0.0518 (15)	0.0070 (13)
C5	0.0591 (13)	0.0448 (12)	0.0642 (14)	0.0004 (10)	0.0376 (12)	0.0015 (10)
C6	0.0505 (12)	0.0457 (12)	0.0474 (12)	0.0001 (10)	0.0283 (11)	0.0022 (9)
C7	0.0525 (13)	0.0397 (11)	0.0467 (12)	−0.0004 (9)	0.0268 (10)	−0.0004 (9)
C8	0.0548 (13)	0.0427 (12)	0.0415 (11)	−0.0009 (9)	0.0270 (10)	0.0009 (9)
C9	0.0759 (16)	0.0490 (13)	0.0541 (13)	−0.0033 (11)	0.0418 (13)	−0.0016 (10)
C10	0.0572 (13)	0.0459 (13)	0.0509 (12)	0.0001 (10)	0.0318 (11)	0.0007 (10)
C11	0.0874 (19)	0.0469 (14)	0.0852 (19)	−0.0087 (12)	0.0561 (16)	0.0020 (12)

### Geometric parameters (Å, º)

**Table:**

O1W—H1W	0.92 (3)	C3—C4	1.368 (4)
N1—C7	1.362 (3)	C4—C5	1.363 (3)
N1—C6	1.409 (3)	C4—H4A	0.9300
N1—H1A	0.88 (3)	C5—C6	1.387 (3)
O1—C7	1.215 (2)	C7—C8	1.478 (3)
F1—C3	1.360 (3)	C8—C10	1.363 (3)
C1—C2	1.384 (3)	C8—C9	1.404 (3)
C1—C6	1.389 (3)	C9—H9A	0.9300
C1—H1B	0.9300	C10—C11	1.475 (3)
F2—C5	1.354 (3)	C11—H11A	0.9600
O2—C10	1.344 (3)	C11—H11B	0.9600
O2—N2	1.406 (3)	C11—H11C	0.9600
N2—C9	1.299 (3)	C11—H11D	0.9600
C2—C3	1.355 (4)	C11—H11E	0.9600
C2—H2B	0.9300	C11—H11F	0.9600
			
C7—N1—C6	126.79 (19)	C4—C5—C6	123.9 (2)
C7—N1—H1A	117.3 (16)	C5—C6—C1	116.6 (2)
C6—N1—H1A	115.8 (16)	C5—C6—N1	117.51 (19)
C2—C1—C6	120.6 (2)	C1—C6—N1	125.85 (19)
C2—C1—H1B	119.7	O1—C7—N1	123.6 (2)
C6—C1—H1B	119.7	O1—C7—C8	121.66 (19)
C10—O2—N2	108.88 (17)	N1—C7—C8	114.70 (18)
C9—N2—O2	105.04 (18)	C10—C8—C9	103.82 (19)
C3—C2—C1	119.3 (2)	C10—C8—C7	125.4 (2)
C3—C2—H2B	120.4	C9—C8—C7	130.7 (2)
C1—C2—H2B	120.4	N2—C9—C8	112.8 (2)
C2—C3—F1	119.4 (3)	N2—C9—H9A	123.6
C2—C3—C4	122.8 (2)	C8—C9—H9A	123.6
F1—C3—C4	117.8 (2)	O2—C10—C8	109.43 (19)
C5—C4—C3	116.7 (2)	O2—C10—C11	116.39 (19)
C5—C4—H4A	121.6	C8—C10—C11	134.2 (2)
C3—C4—H4A	121.6	H11D—C11—H11E	109.5
F2—C5—C4	118.5 (2)	H11D—C11—H11F	109.5
F2—C5—C6	117.62 (19)	H11E—C11—H11F	109.5
			
C10—O2—N2—C9	1.0 (3)	C6—N1—C7—O1	−3.4 (4)
C6—C1—C2—C3	−0.2 (4)	C6—N1—C7—C8	176.0 (2)
C1—C2—C3—F1	−179.4 (3)	O1—C7—C8—C10	−13.8 (3)
C1—C2—C3—C4	0.3 (5)	N1—C7—C8—C10	166.8 (2)
C2—C3—C4—C5	0.6 (4)	O1—C7—C8—C9	162.3 (2)
F1—C3—C4—C5	−179.7 (3)	N1—C7—C8—C9	−17.1 (3)
C3—C4—C5—F2	178.2 (2)	O2—N2—C9—C8	−0.6 (3)
C3—C4—C5—C6	−1.6 (4)	C10—C8—C9—N2	0.0 (3)
F2—C5—C6—C1	−178.2 (2)	C7—C8—C9—N2	−176.8 (2)
C4—C5—C6—C1	1.6 (4)	N2—O2—C10—C8	−1.0 (3)
F2—C5—C6—N1	1.3 (3)	N2—O2—C10—C11	178.9 (2)
C4—C5—C6—N1	−178.9 (2)	C9—C8—C10—O2	0.6 (2)
C2—C1—C6—C5	−0.7 (4)	C7—C8—C10—O2	177.6 (2)
C2—C1—C6—N1	179.9 (2)	C9—C8—C10—C11	−179.2 (3)
C7—N1—C6—C5	−169.5 (2)	C7—C8—C10—C11	−2.2 (4)
C7—N1—C6—C1	9.9 (4)		

### Hydrogen-bond geometry (Å, º)

**Table:**

*D*—H···*A*	*D*—H	H···*A*	*D*···*A*	*D*—H···*A*
N1—H1*A*···O1*W*	0.88 (3)	2.26 (3)	3.052 (3)	150 (2)
O1*W*—H1*W*···N2^i^	0.92 (3)	2.03 (3)	2.934 (2)	167 (3)

Symmetry code: (i) *x*, −*y*+1, *z*−1/2.
